# Panniculitis with late onset enthesitis-related arthritis: a case report

**DOI:** 10.1186/s12969-023-00888-7

**Published:** 2023-09-11

**Authors:** Wenxiu Mo, Fei Sun, Tongxin Han, Huawei Mao

**Affiliations:** Department of Immunology, Ministry of Education Key Laboratory of Major Diseases in Children, Beijing Children’s Hospital, Capital Medical University, National Center for Children’s Health, 56 South Lishi Road, Xicheng District, Beijing, China

**Keywords:** Panniculitis, Enthesitis-related arthritis, Extra-articular manifestation, Anti-TNF-α

## Abstract

**Background:**

Panniculitis, a type of inflammation of subcutaneous fat, is a relatively uncommon condition that usually presents as inflammatory nodules or plaques, with various proposed etiologic factors. The association between panniculitis and enthesitis-related arthritis has not been described previously.

**Case presentation:**

Herein, we describe a case of a 11-year-old girl who presented with recurrent fever and painful subcutaneous nodules on her extremities and buttocks. Histological examination of the skin biopsy specimen revealed lobular panniculitis. Despite the use of prednisone and mycophenolate mofetil for several months, the patient experienced a relapse of skin lesions and additional symptoms of peripheral joint swelling and inflammatory lumbar pain. She was diagnosed with enthesitis-related arthritis after confirmation by imaging. The panniculitis demonstrated a sustained response when a tumor necrosis factor alpha inhibitor was used for enthesitis-related arthritis. At 2-year follow-up, her skin lesions and arthritis remained stable.

**Conclusions:**

Although rare, panniculitis can be considered an unusual extra-articular manifestation of enthesitis-related arthritis based on clinical and pathological insights.

## Introduction

Panniculitis is a type of inflammation of subcutaneous fat, and is a relatively uncommon condition that usually presents as inflammatory nodules or plaques, often accompanied by episodic fever, chills, and myalgia. It can be related to infections (streptococcal infection or tuberculosis), external insults (traumatic or cold panniculitis), malignancy, or connective tissue diseases (CTD), such as systemic lupus erythematosus (SLE) and dermatomyositis (DM).

Enthesitis-related arthritis (ERA) is a type of juvenile idiopathic arthritis (JIA) that belongs to the spectrum of diseases in juvenile spondyloarthropathy. Although ERA is characterized by a greater frequency of extra-articular manifestations in children, including uveitis and inflammatory bowel disease (IBD), than in adults with spondyloarthropathy [1], the association of skin lesions and ERA has not previously been described. Herein, we report the first case of ERA-associated panniculitis, which may provide clinical and pathological insights.

## Case presentation

**Fig. 1:**
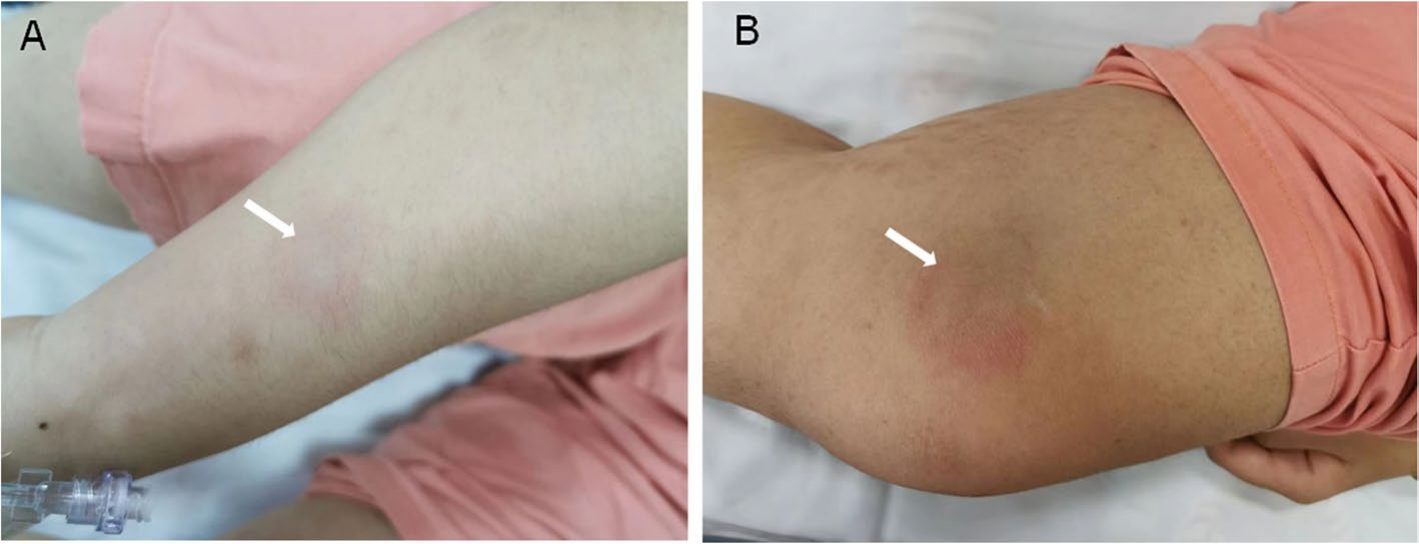
Gross appearance of nodules. Multiple erythema with tenderness on patient's upper (**A**) and lower (**B**) limbs

A 11-year-old girl presented with recurrent fever and erythema for 2 months. Her medical history included episodes of fever, accompanied by painful subcutaneous nodules on her extremities and buttocks. The patient initially responded well to treatment with dexamethasone (5 mg, intramuscular injection) instead of antibiotics; however, she experienced symptomatic relapse approximately 2 weeks later. Physical examination revealed multiple tender erythematous nodules in the upper and lower extremities (Fig. 1A, B). Physical examination results were otherwise unremarkable.

Complete blood count (CBC) showed normal levels of hemoglobin and platelets, with slightly elevated leukocytes (13.01 × 10^9^/L with 87.5% neutrophils). High acute phase reactants, C-reactive protein (CRP) (32.7-105 mg/L; normal < 10 mg/L) and erythrocyte sedimentation rate (ESR) (36-53 mm/h; normal < 20 mm/h), were observed.

A detailed clinical assessment was performed to identify the risk factors for panniculitis, including anti-streptolysin O (ASO), T-SPOT.TB, tuberculin purified protein derivative test, blood culture, antinuclear antibody (ANA), anti-double-stranded DNA (anti-dsDNA), extractable nuclear antigen, perinuclear and cytoplasmic anti-neutrophil cytoplasmic antibody (p-ANCA and c-ANCA), antiphospholipid antibodies, lupus anticoagulant, serum levels of C3 and C4, and alpha 1-antitrypsin were detected, and all were negative or within the normal range. Abdominal ultrasonography and computed tomography (CT) of the abdomen revealed no abnormalities.

**Fig. 2:**
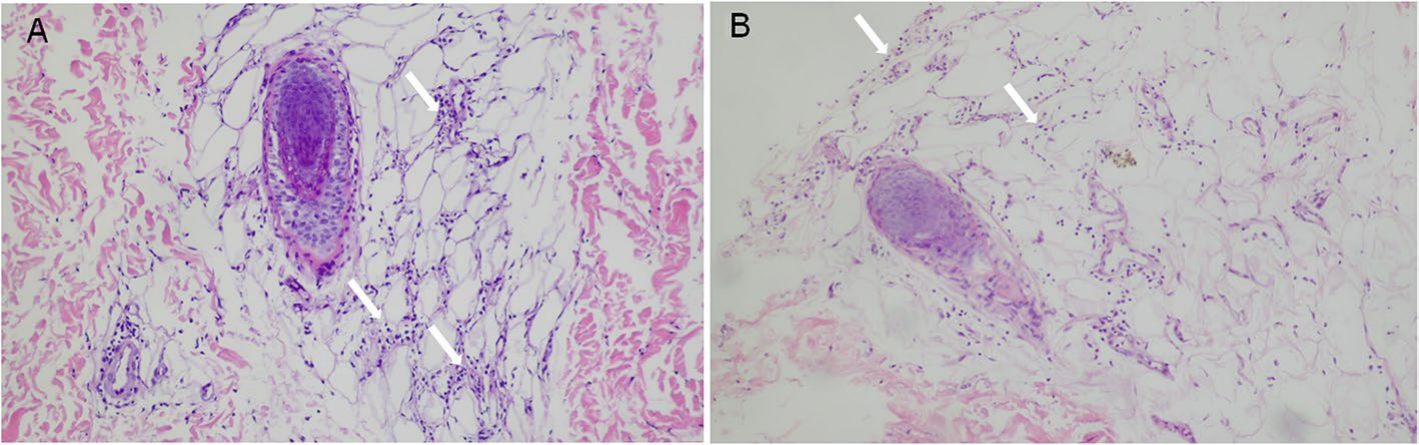
Histopathological staining of skin nodules. Hematoxylin–eosin, original magnification (200 ×) showed moderate (**A**) and small (**B**) infiltration of lymphocytes and histiocytes in fat lobules for the first and second time biopsy

Histopathological examination of the skin biopsy specimen obtained from a representative nodule revealed lobular panniculitis with moderate infiltration of lymphocytes and histiocytes (Fig. 2A). No evidence of vasculitis, necrosis, or infection caused by bacteria, fungi, or mycobacteria was observed in the biopsy specimen. Based on the present case report and a combination of serological tests and histopathological examination, we diagnosed the patient with panniculitis.

**Fig. 3:**
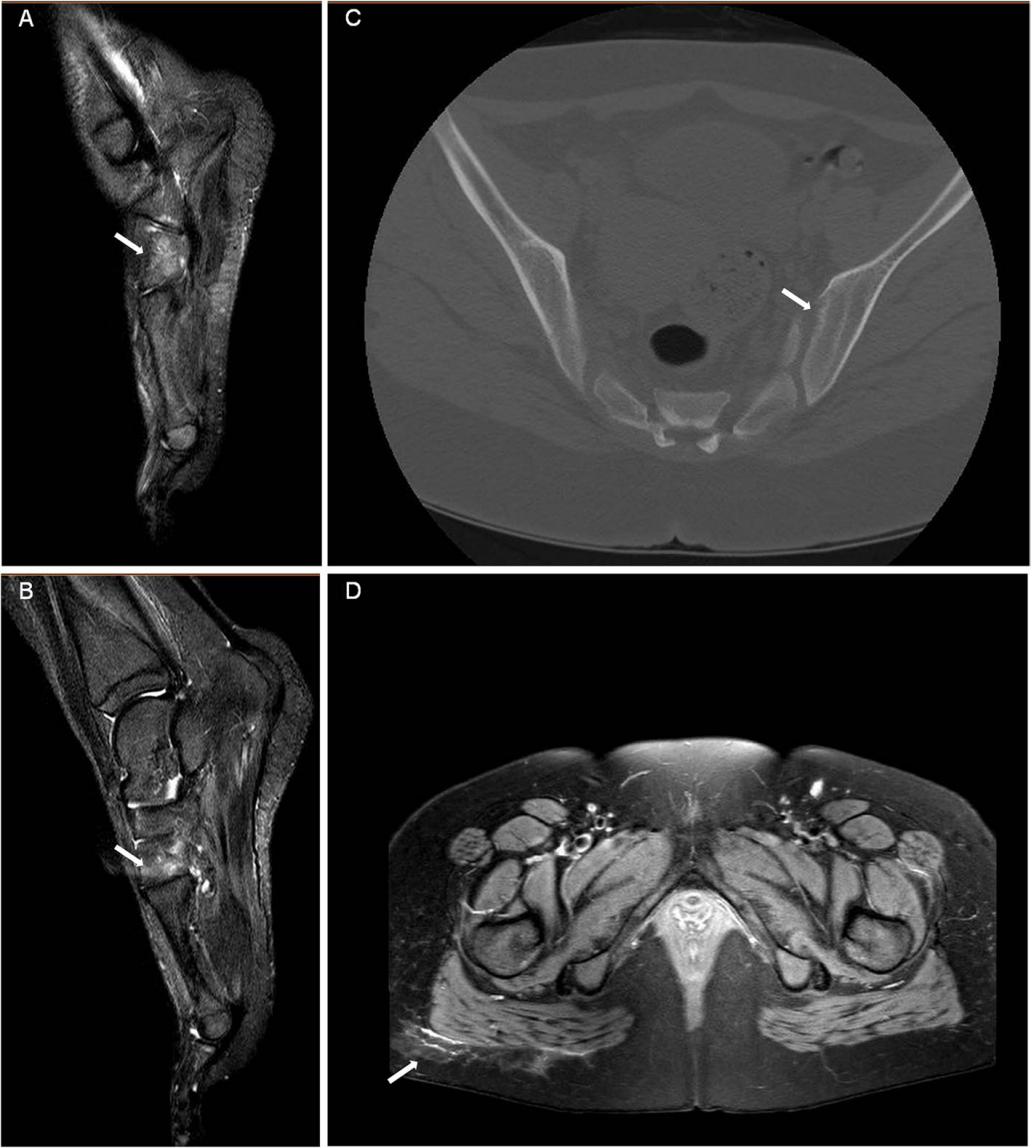
Imagines of left foot, Sacroiliac joint and hip. MRI of left foot showed middle foot involvement with bone marrow oedema in cuboid (**A**) and cuneiform (**B**); SIJ CT revealed sacroiliitis with bone destruction (**C**); MRI of hip showed inflammation in fat layer at the same time (**D**)

The patient received prednisone (1.5 mg/kg/day) combined with mycophenolate mofetil (MMF) (300 mg/m^2^/dose) as glucocorticoid-sparing therapy which showed obvious improvement. However, with prednisone tapered to 10 mg/day for approximately 10 months, the girl suffered again from high fever and tenderness nodular, along with new symptoms: her left ankle and foot became swollen and there was point tenderness on the dorsal side of the talonavicular joint. Her medical history revealed no history of acute anterior uveitis or IBD. Laboratory examination results, including those for ASO, human leukocyte antigen 27 (HLA-B*27), rheumatoid factor, anti-cyclic citrullinated peptide, and ANA, were negative. Magnetic resonance imaging (MRI) of the left foot showed that the tarsal area involvement with bone marrow edema in the cuboid and cuneiform bones (Fig. 3A, B), indicating enthesitis. A second skin biopsy revealed a small aggregation of lymphocytes and histiocytes in the fat lobules (Fig. 2B). Therefore, prednisone treatment was increased to 15 mg/day, and ibuprofen (7.5 mg/kg/dose) was prescribed as well. However, the patient had recurrent episodes of painful erythema and swelling in her left foot, with gradual low back pain 1 year before admission. CT of the sacroiliac joint (SIJ) revealed sacroiliitis with bone destruction (Fig. 3C) and the hip MRI showed inflammation in the fat layer (Fig. 3D).

**Fig. 4:**
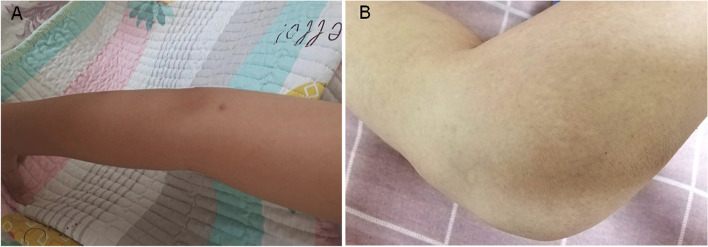
Gross appearance of patient's extremities after treatment. A relief of erythema on patient's upper (**A**) and lower (**B**) limbs

**Fig. 5:**
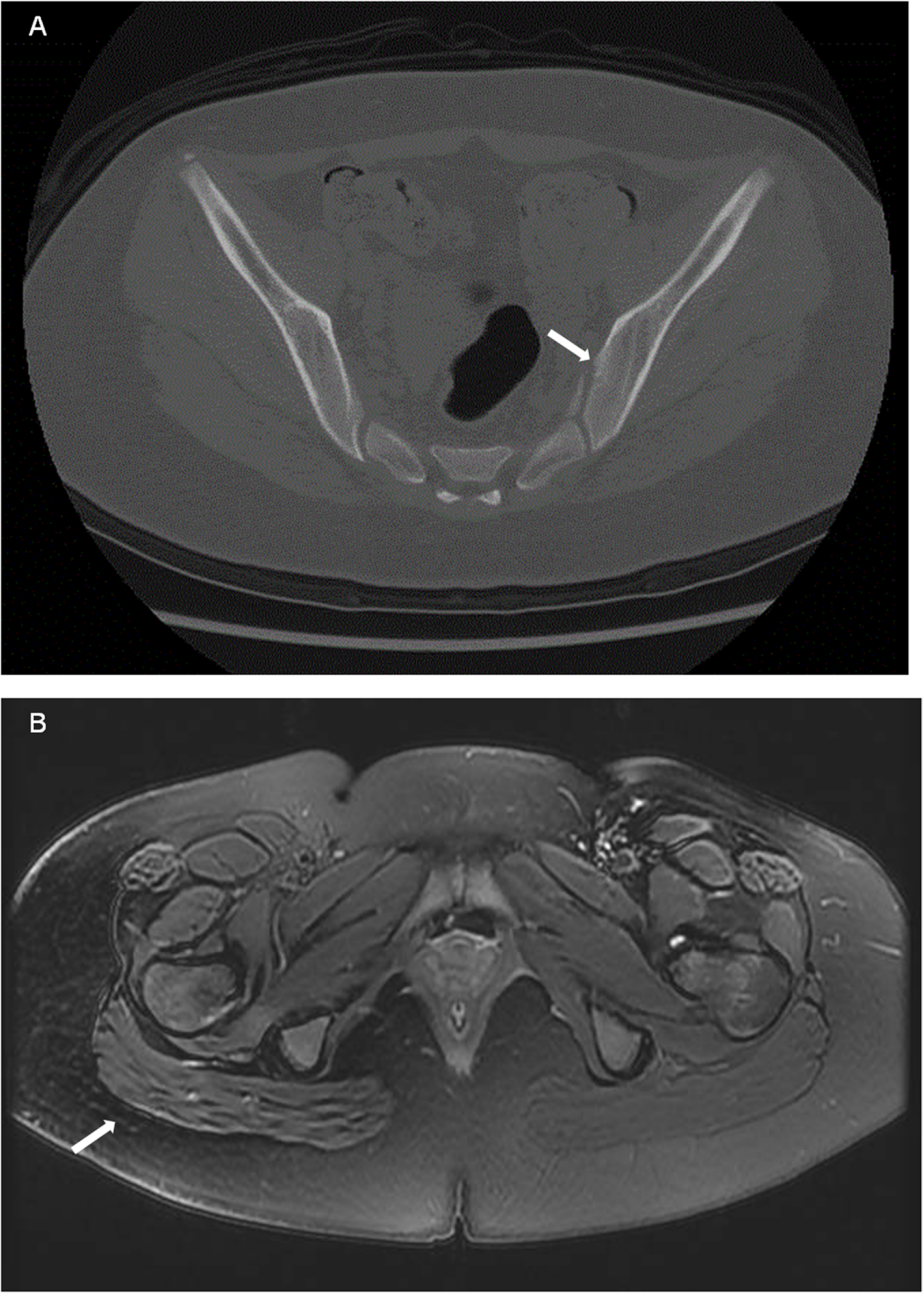
Imagines after treatment. CT revealed no further progression of bone destruction in the SIJ (**A**), and MRI of hip showed inflammation of the fat layer on the buttocks was significantly reduced (**B**)

Finally, the patient was diagnosed with panniculitis and ERA, with the latter diagnosed based on the International League of Associations for Rheumatology classification criteria for JIA 2001 [2]. Anti-tumor necrosis factor alpha (anti-TNF-α) antibody infliximab (IFX) (5 mg/kg at 2-week intervals initially, repeated in monthly intervals three times, currently at 8-week intervals) plus methotrexate (15 mg/m^2^) was chosen as therapy, whereas prednisone and MMF were gradually discontinued. She experienced an improvement in both joints and panniculitis (Fig. 4A, B). Laboratory tests showed that CBC and serum acute phase reactants returned to the normal range. Furthermore, CT revealed no further progression of bone destruction in the SIJ (Fig. 5A), and inflammation of the fat layer on the buttocks was significantly reduced (Fig. 5B). At 2-year follow-up, her skin lesions and arthritis remained stable.

## Discussion

Panniculitis is an uncommon condition with a multifactorial etiology. It can be a skin complication of various autoimmune diseases, including SLE, DM and vasculitis [3]. However, few studies have reported panniculitis with arthritis.

In this case, the patient presented with recurrent painful erythema, verified by histopathological examination as lobular panniculitis. On histological examination, the predominance of lymphocytes and histiocytes, rather than neutrophilic cells, indicated that neutrophilic lobular panniculitis associated with rheumatoid arthritis (RA) and Crohn's disease could be largely ruled out. Additionally, the absence of vasculitis excluded the possibility of nodular vasculitis (Bazin disease) and some infectious panniculitis, such as leprosy nodular erythema. Considering the patient’s clinical features, including age, medical history, marked inflammatory state with high fever, and elevated inflammatory markers, lobular panniculitis associated with systemic disease was strongly suspected, including SLE, JDM, pancreatic disease, and potentially some neoplastic diseases.

Skin lesions are considered idiopathic or a cutaneous manifestation of an unknown systemic disease without positive findings at that time. However, we could not wait until the "clinical picture" was fully displayed; therefore, glucocorticoid was prescribed and tapered with careful attention. Meanwhile, we repeatedly screened for underlying systemic diseases until the presence of arthritis and ERA was confirmed by imaging.

Regarding disease progression, panniculitis with high inflammation precedes the development of overt arthritis, and it was poorly controlled with glucocorticoid tapering, which also could not effectively prevent progression of arthritis. Therefore, we diagnosed the patient with panniculitis with late-onset ERA. The patient reported considerable relief of joint and back pain with anti-TNF-α therapy, and no fever or skin manifestation recurrence was observed at the 2-year follow-up. Hence, the association between panniculitis and ERA has been discussed here from the perspective of disease development and therapy.

ERA is a specific category of JIA that is characterized by axial or peripheral arthritis and enthesitis. Different extra-articular manifestations can encompass the clinical spectrum. These include severe muscular atrophy, growth failure, pubertal delay, ophthalmological complications, IBD, and psoriasis [4]. Although rare, SAPHO (synovitis, acne, pustulosis, hyperostosis, osteomyelitis) syndrome [5], secondary renal amyloidosis [4, 6], and cardiac involvement, such as aortitis [7] have also been reported in ERA. However, skin disorders have been seldom reported, and no cases of panniculitis have been previously reported.

On the other hand, in most cases, panniculitis presents with episodes of joint pain and swelling due to acute inflammatory effusion; however, imaging-confirmed persistent arthritis is uncommon [8]. Few cases of panniculitis and arthritis have been reported worldwide. Of these, panniculitis occurs together with arthritis in some patients to form a triad of pancreatitis, panniculitis, and polyarthritis, referred to in the literature as PPP syndrome [9], which is associated with acute and chronic pancreatitis and pancreatic malignancies. Owing to the presence of pancreatic disease, panniculitis, and arthritis were considered secondary to fat necrosis. Two other cases reported mycobacterium-associated lobular panniculitis that mimicked a rheumatoid nodule in RA [10] and a panniculitis with RA without obvious infection [11]. However, unlike these studies, our patient showed no evidence of pancreatic involvement or pathogenic infection. Therefore, we believe that panniculitis with late-onset ERA is a unique condition.

Studies on possible mechanisms underlying this association have also been reported. In 2015, Dennis McGonagle et al. proposed a concept of "MHC-I-opathy," including Behçet disease and several clinically distinct spondyloarthropathies—all associated with MHC-I alleles, such as HLA-B*51, HLA-B*27, HLA-C*0602, or epistatic endoplasmic reticulum aminopeptidase 1(ERAP1) interaction [12]. These disorders share key features of disease localization to physical stress sites, either at barrier surfaces (oral mucosa, gut, and skin) or internal sites (joints and entheses, including interfaces between mobile structures in the eye, vessel walls, and valves). An abnormal response to normal levels of stress or a normal response to abnormal levels of stress occurring at barrier surfaces or sites of microdamage collectively lead to the priming of innate immunity. That was just like we observed the association between IBD and its extra-intestinal manifestations, which involve the mouth, skin (barrier surfaces), and joints, eyes (interfaces between mobile structures). Therefore, immunologically, spondyloarthropathies such as ERA and skin abnormality panniculitis may share a common immunopathogenetic basis. Based on the perspective of monism, ERA with panniculitis may involve different presentations of "MHC-I-opathy".

Another evidence that verifies the entity of ERA with panniculitis is the high efficacy of anti-TNF-α therapy. IFX has been used to treat orbital lobular panniculitis [13] and other inflammatory dermatoses [14] with satisfactory results. TNF-α is an inflammatory cytokine released by many cell types, including monocytes, macrophages and lymphocytes, which are major cells contributing to panniculitis. High-affinity receptors of TNF-α were also expressed in adipose tissue [15]. Therefore, treating panniculitis with anti-TNF-α agents that are equally effective for ERA is reasonable. And our patient experienced a clear and sustained benefit from this approach.

Paradoxically, TNF-α blockade can lead to psoriasis and erythema nodosum because of a dysregulated type I interferon response [16]. Specifically, anti-TNF treatment prolongs type I interferon production by plasmacytoid dendritic cells by inhibiting their maturation. As this patient received TNF-α antagonist therapy, this should be seriously considered. The skin lesions in this patient require further investigation.

## Conclusions

From clinical and pathological insights, although rare, skin lesions, such as panniculitis, should be considered as possible extra-articular manifestations when ERA is defined. Furthermore, this indicates that many skin lesions, although not specific, require further investigation to identify a possible underlying inflammatory rheumatic disease, and unusual extra-articular manifestations of arthritis can respond positively to proper treatment and management.

## Data Availability

Not applicable.

## Abbreviations

ANA: Antinuclear antibody
ASO: Anti-streptolysin O
anti-dsDNA: Anti-double-strand DNA
anti-CCP: Anti-cyclic citrullinated peptide
anti-TNF-α: Anti-tumor necrosis factor TNF alpha
CRP: C-reactive protein
CT: Computerized tomography
CTD: Connective tissue disease
c-ANCA: Cytoplasmatic anti-neutrophil cytoplasmic antibody
DM: Dermatomyositis
ENA: Extractable nuclear antigen
ERA: Enthesitis-related arthritis
ESR: Erythrocyte sedimentation rate
ERAP1: Endoplasmic reticulum aminopeptidase 1
HLA-B*27: Human leukocyte antigen 27
IBD: Inflammatory bowel disease
IFX: Infliximab
JIA: Juvenile idiopathic arthritis
MMF: Mycophenolate mofetil
MRI: Magnetic resonance imaging
MTX: Methotrexate
pDCs: Plasmacytoid dendritic cells
PPD: Purified protein derivative
p-ANCA: Perinuclear anti-neutrophil cytoplasmic antibody
RF: Rheumatoid factor
SAPHO: Synovitis, acne, pustulosis, hyperostosis, osteomyelitis
SLE: Systemic lupus erythematosus
SIJ: Sacroiliac joint
